# Exploring a Novel Hypothesis: Could the Eye Function as a Radar or Ultrasound Device in Depth and Distance Perception? Neurophysiological Insights

**DOI:** 10.3390/life15040536

**Published:** 2025-03-25

**Authors:** Hüseyin Findik, Muhammet Kaim, Feyzahan Uzun, Ayhan Kanat, Osman Nuri Keleş, Mehmet Dumlu Aydin

**Affiliations:** 1 Department of Ophthalmology, School of Medicine, Recep Tayyip Erdogan University, 53100 Rize, Turkey; muhammet.kaim@erdogan.edu.tr (M.K.);; 2 Department of Neurosurgery, School of Medicine, Recep Tayyip Erdogan University, 53100 Rize, Turkey; 3 Department of Histology, School of Medicine, Ataturk University, 25030 Erzurum, Turkey; 4 Department of Neurosurgery, School of Medicine, Ataturk University, 25030 Erzurum, Turkey; nmda11@hotmail.com

**Keywords:** retina, radar, ultrasound, depth perception, distance perception, neurophysiology, visual system

## Abstract

Recent advancements in ocular physiology suggest that the eyes may function similarly to radar antennae or ultrasound probes, with the occipital cortex acting as a detector, challenging the traditional view of binocular vision as the primary mechanism for depth and distance perception. Methods: We conducted a comprehensive analysis of the neuroanatomical and histological architecture of the neuro-optico-cortical systems in a male wild rabbit model. The objective was to identify potential structural and functional similarities between the retino-optical system and radar/ultrasound effector-detector systems. Results: Histological examination revealed significant similarities between retinal morphology and radar/ultrasound systems. The outermost retinal layer resembled an acoustic lens, with underlying layers functioning as acoustic matching layers. The ganglion cell layer exhibited characteristics akin to the piezoelectric elements of transducers. Conclusions: Our findings support the hypothesis that the retinal apparatus functions similarly to radar antennae or ultrasound probes. Light-stimulated retinal-occipital cortex cells perceive objects and emit electromagnetic waves through the retina, which are reflected by objects and processed in the occipital cortex to provide information on their distance, shape, and depth. This mechanism may complement binocular vision and enhance depth and distance perception in the visual system. These results open new avenues for research in visual neuroscience and could have implications for understanding various visual phenomena and disorders.

## 1. Introduction

The evaluation of distance and depth perception is fundamental to human visual processing, playing a critical role in everyday activities. Traditionally, these abilities have been attributed to the integration of binocular vision with neuro-optical networks [1,2]. However, emerging evidence suggests the existence of alternative mechanisms, which may expand our understanding of how the visual system operates.

The study of visual physiology and perceptual mechanisms has a long and rich history, originating in antiquity [3,4]. Alcmaeon of ancient Greece hypothesized that vision arose from optic nerves channeling fire encased in water within the eyes [5]. Democritus suggested that objects emitted atom-like particles perceived as colors, while Hippocrates examined the anatomical structure of the eye to uncover its functional rationale [6]. During Galen’s time, vision was believed to be mediated by the eyes as effector organs, relying on pneuma derived from brain ventricles [7,8]. Advances during the Renaissance unveiled the anatomical basis of vision, including the optic chiasm, optical radiation, and the laminar organization of the occipital lobes [9]. In modern times, the visual system has been demonstrated to function akin to a photomultiplier, with binocular vision identified as essential for depth and distance perception [10]. Moreover, studies have shown that the eyes generate electromagnetic waves similar to brain waves [11], and the light-stimulated retina produces resting potentials of 6–10 mV [12,13].

Despite these advances, alternative mechanisms for depth and distance perception have garnered growing interest. Beyond the conventional role of binocular vision, monocular cues such as motion parallax, size constancy, texture gradients, and occlusion significantly contribute to depth perception, particularly when binocular input is limited or absent [14]. The brain’s remarkable adaptability in integrating these monocular cues underscores the complexity of the visual system [15].

Recent findings in visual neuroscience suggest that the retina and visual pathways may process information through mechanisms beyond classical optical theories. Specifically, electromagnetic wave interactions and bioelectric signaling within the retina raise the possibility that it may encode spatial and distance information in a manner analogous to radar or sonar systems [16,17].

Another fascinating avenue involves neural oscillations and synchrony within the occipital cortex [18]. Gamma-band activity and phase synchronization have been implicated in the brain’s ability to integrate spatial and temporal information, potentially contributing to depth and distance encoding [19]. Additionally, proprioceptive and vestibular inputs may complement visual cues, creating an integrated sensory framework for spatial awareness [20,21]. This multimodal integration enables robust depth perception even in complex, dynamic environments.

Building on these principles, this study proposes a novel hypothesis that the retina may function similarly to radar antennas or ultrasound probes, with the occipital cortex processing reflected signals. In this model, light-stimulated retinal cells could emit electromagnetic waves toward surrounding objects, while the visual system analyzes the returning waves to aid in depth and distance perception. Through neurophysiological and histological approaches, this investigation examines structural and functional parallels between the retinal apparatus and radar or ultrasound systems.

The findings from this study may transform our understanding of the visual system, emphasizing its intricate network that integrates diverse physiological and neural processes to achieve depth and distance perception. This perspective could pave the way for new research directions in visual physiology, neuro-ophthalmology, and biomedical engineering, with significant implications for the understanding and treatment of visual disorders.

## 2. Materials and Methods

This study was conducted on a male wild rabbit model to analyze the anatomical and histological network architectures of the neuro-opticocortical system and investigate potential structural similarities between the retino-optical system and radar-ultrasound effector-detector systems.

Animal protocol was reviewed and approved by the Ethics Committee of Erzurum Atatürk University, Faculty of Medicine. The care of the animal model and the experimental procedures adhered to the guidelines established by the ethics committee to ensure humane treatment and ethical compliance. To minimize pain and mortality during the study, a balanced injectable anesthetic protocol was utilized. Anesthesia was initially induced with isoflurane administered via a face mask. Subsequently, a combination of ketamine hydrochloride (150 mg/1.5 mL), xylazine hydrochloride (30 mg/1.5 mL), and distilled water (1 mL) was prepared, and a dose of 0.2 mL/kg was administered subcutaneously prior to surgical procedures. During the experiment, an additional dose of 0.1 mL/kg of the same anesthetic combination was provided as required to maintain adequate anesthesia. Post-mortem analysis of the retinal and optical tissues was performed to obtain detailed histological and anatomical data.

### Histopathological Procedures

To estimate neuronal density in the retina, bilateral retinal tissues were carefully excised. The specimens were horizontally embedded in paraffin blocks to ensure optimal visualization of all retinal layers during histopathological examination. Sections were stained using hematoxylin and eosin (H&E) (Merck GmbH, Darmstadt, Germany) and Masson’s trichrome (MTC) (Sigma-Aldrich, St. Louis, MI, USA) stains to facilitate detailed morphological analysis.

Neuronal density was assessed using the physical dissector method, a reliable stereological technique. This method offers several advantages: it provides unbiased estimates of particle number, is straightforward to perform, does not rely on assumptions about particle shape, size, or orientation, and is unaffected by overprojection or truncation artifacts. Two consecutive sections (referred to as dissector pairs) were obtained from the tissue samples and mounted on each slide. The reference and look-up sections were alternated to effectively double the number of dissector pairs without the need for additional tissue sections.

Coronal sections of the ocular bulb, taken at the level of the ocular equator, were also prepared for analysis. These sections were stained with H&E to further investigate the structural and histological characteristics of the eye.

## 3. Results

### 3.1. Histological Analysis of the Retina

**Figure 1:**
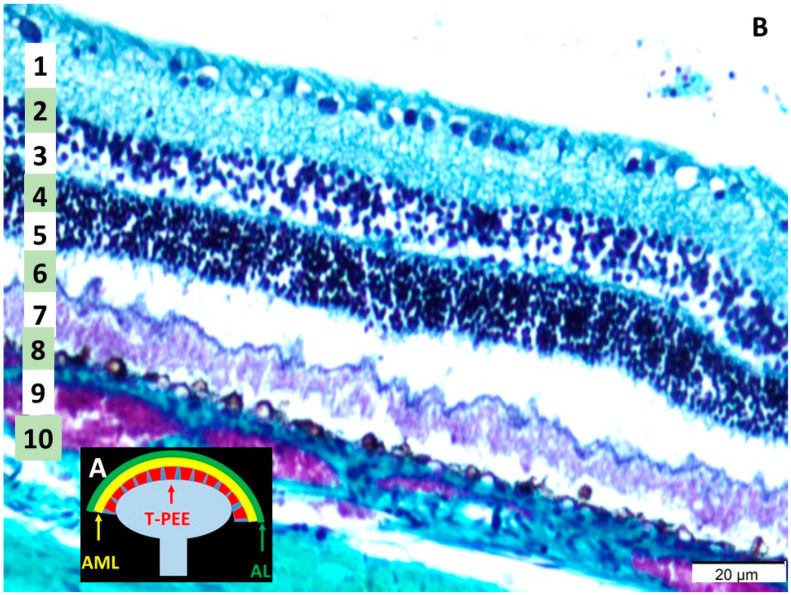
(**A**) Acoustic lens (AL) in the probe of an ultrasound device, transducer of piezoelectric transducer (T-PEE) and the acoustic matching layer (AML) (**B**) Histological appearance of choroid and retinal layers: (1) the inner limiting membrane, (2) the nerve fiber layer, (3) the ganglion cell layer, (4) the inner plexiform layer, (5) the inner nuclear layer, (6) the outer plexiform layer, (7) the outer nuclear layer, (8) the outer limiting membrane, (9) the photoreceptor layer, and (10) the retinal pigmented epithelium (LM, MTC, ×40).

The histological structure of the retina, optic nerve, and retinal neurons was examined in detail, as shown in Figure 1. The retina consists of 10 distinct layers, as described before: (1) inner limiting membrane (ILM), (2) nerve fiber layer (NFL), (3) ganglion cell layer (GCL), (4) inner plexiform layer (IPL), (5) inner nuclear layer (INL), (6) outer plexiform layer (OPL), (7) outer nuclear layer (ONL), (8) outer limiting membrane (OLM), (9) photoreceptor layer (PL), and (10) the retinal pigmented epithelium (RPE) [22]. Notably, morphological similarities were identified between the retinal structure and the functional components of radar antennas and ultrasound probes. The outermost layer of the retina resembled the acoustic lens of these systems, while the underlying layer was analogous to the acoustic matching layer. Furthermore, the ganglion cell layer displayed structural characteristics akin to the piezoelectric elements of a transducer, which are essential for converting signals in radar and ultrasound systems.

### 3.2. Retinal Function and Radar Analogy

**Figure 2:**
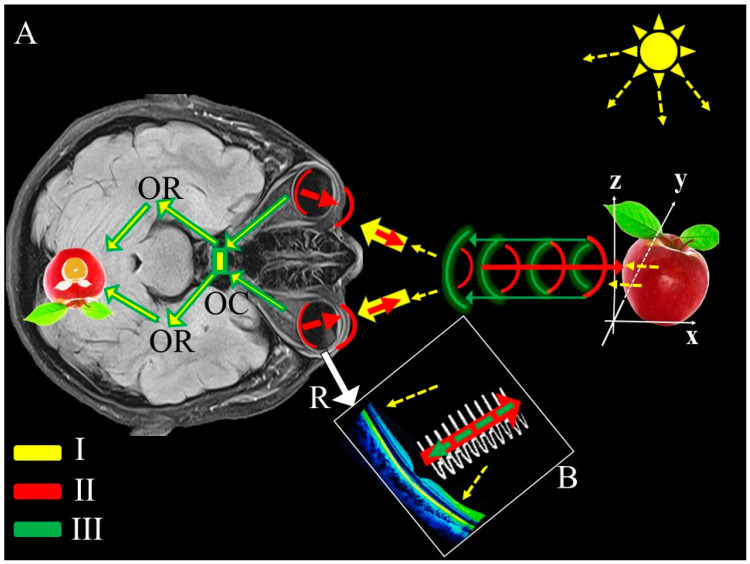
In the figure, a schematic summary explaining the formation of the sense of distance and depth according to our hypothesis is observed. In part (**A**); as a result of the rays emitted from the sun and hitting the apple and reaching the eye, passing through the retina (R), optic chiasm (OC), and optic radiation (OR) and reaching the occipital cortex, which is the visual field (yellow arrows), we first only notice the apple (yellow arrows). At this time, the electromagnetic waves (White wave beam (**B**)), formed at a value of 6–10 mV in the retina (R) stimulated by light, are spread from the retina, which is like a radar dish, to the external environment. In this way, the electromagnetic waves (Red arrows and bows (**A**,**B**)) leaving the retina and leaving the pupil hit the object and return (Green arrows and bows), while a certain amount of energy and time loss occurs depending on the characteristics of the external and internal environments. Neural networks, especially those located in the retina and occipital cortex and working like the signal processing chips and screens of radars, inform the consciousness about the distance, proximity, and depth of objects by taking advantage of the time and energy differences of the first and last signals that come to them. The order of emergence and flow of the rays involved in the formation of vision and depth is symbolized in the lower left corner of the picture.

As illustrated in Figure 2, the functional analogy between the retina and radar systems was conceptualized using principles of reflected power. The reflected power (Pr) at the retina is influenced by factors such as the power density (Su), the antenna gain (G), and the variable retinal cross-section (σ). This relationship can be expressed as:

Su × ς × G/R^2^_1_

Pr = reflected power (WC = retinal cross-section (mm^2^), R_1_ = range, distance retina-to target (m).

In this analogy, the target can be regarded as a secondary radiator, reflecting power back to the retina. The reflected power serves as the emitted signal that the retina processes.

### 3.3. Comparative Observations

Overall, the analysis revealed striking parallels between retinal morphology and the structural–functional elements of radar antennas and ultrasound probes. The outermost retinal layer functions analogously to an acoustic lens, focusing incoming signals, while the acoustic matching layer is mirrored in the subsequent layers. The ganglion cell layer exhibits functional similarities to piezoelectric elements, crucial for signal transduction. These findings highlight the potential for the retina to operate as an integrated biological system capable of complex signal processing, akin to engineered radar and ultrasound systems.

## 4. Discussion

This study investigated the hypothesis that the eyes might function analogously to radar or ultrasound devices, yielding compelling evidence in support of this concept. The findings highlight remarkable structural similarities between the retina and the components of radar antennas and ultrasound probes, reinforcing the premise that the eyes may possess the capacity to emit electromagnetic waves. Histological analysis of the retinas from a male rabbit model revealed morphological structures that closely parallel those found in these advanced technologies. Specifically, the outermost retinal layer exhibits similarities to an acoustic lens, the underlying layer resembles an acoustic coupling layer, and the ganglion cell layer shares structural features with piezoelectric transducer elements (Figure 1). These observations suggest that the retina’s unique architecture may facilitate both the emission and detection of electromagnetic waves, providing a novel perspective on ocular function.

Wang et al. [23] presented groundbreaking research that offered a novel perspective on the electromagnetic properties of the visual system, demonstrating that the eye produces biophoton emissions. This discovery suggests that the retina is not merely a light-absorbing structure but also actively emits electromagnetic radiation. When considered alongside the intricate layered architecture of the retina and the potential electromagnetic properties of each layer, this phenomenon lends support to the hypothesis that the eye may possess radar- or ultrasound-like functional capabilities [24].

Uncertainties surrounding the coherence length and directional emission properties of biophotons raise questions about their suitability for spatial distance perception. However, recent findings indicate that biophotons contribute significantly to visual processing. Studies suggest that neuronal oscillations and phase correlations may enable biophotons to enhance contrast mechanisms in the retina and modulate optical feedback [23,25,26]. Moreover, the ordered alignment of Müller cells within the retina may serve as an intrinsic waveguide, facilitating the directional propagation of biophotons and enhancing their interactions with external light sources [27]. The ordered arrangement of photoreceptors could optimize the propagation of biophotons by concentrating their emission in specific directions [26]. Additionally, the retina’s neural network supports visual processing by detecting and integrating weak biophoton signals [28]. To further investigate these mechanisms, experimental approaches utilizing ultra-sensitive photonic detection systems and artificial retina models are recommended [29,30,31]. The integration of biophotons into the brain’s sensory processing system unveils a novel paradigm for depth perception, transcending traditional optical models.

The similarities between retinal morphology and radar and ultrasound systems provide a novel perspective on the functioning of the visual system. By examining the layers and functions of the retina, the hypothesis can be reassessed as follows: the RPE enhances image quality by preventing the back-reflection of light. Strauss [32] highlighted the high metabolic activity of this layer and its critical role in the phagocytosis of photoreceptor outer segments. This functionality is analogous to radar-absorbing materials (RAM) in radar systems, as both mechanisms effectively minimize unwanted reflections. Furthermore, the high metabolic activity and phagocytic processes of the RPE may contribute to the emission of biophotons [23,33]. Regarding the photoreceptor layer, photoreceptors undergo conformational changes in response to light stimuli [34,35] and play a critical role in converting light into electrical signals. This function is analogous to the role of antenna elements in radar systems, which detect electromagnetic waves and transform them into electrical signals. This analogy aligns with the well-established optical properties of the retina, as demonstrated in various studies [36,37,38]. The outer and inner nuclear and plexiform layers of the retina facilitate initial signal processing and transmission. Molnar et al. [39] demonstrated that the DNA structure within cell nuclei possesses the ability to absorb and emit electromagnetic waves. These retinal layers can be compared to signal processing circuits in radar systems, as both filter, amplify and prepare received signals for subsequent processing. This analogy is consistent with findings from studies on retinal signal processing mechanisms [40,41]. The ganglion cell layer comprises the primary neurons responsible for transmitting retinal signals to the brain. Kastner and Baccus [42] demonstrated that ganglion cells are sensitive to magnetic fields. The function of this layer can be compared to the output layer in radar systems. The ability of ganglion cells to process multiple streams of information is further supported by studies conducted by Escobar et al. [43] and Gollisch and Meister [37]. These findings support the hypothesis that ganglion cells may possess the capability to generate and/or sense electromagnetic waves. Additionally, the retinal nerve fiber layer can be likened to data transmission lines in radar systems, as both facilitate the transmission of processed signals to a central processing unit—be it the brain or a computer.

Electro-oculographic (EOG) measurements capture the electrical activity arising from the corneo-retinal potential difference. Due to the dipole properties of the eyeball (positive charge at the cornea and negative charge at the retina), changes in the electrical field during eye movements result in electromagnetic wave emissions, as predicted by Maxwell’s equations. The observed linear correlation between eye movements and electromagnetic wave emissions in EOG measurements suggests that modulation of the corneo-retinal potential influences electromagnetic wave propagation [44,45,46]. This finding, combined with the biophoton emissions arising from the metabolic activity of the RPE and its electromagnetic properties, supports the hypothesis that the eye may function analogously to a radar system. These results align with the extensive research conducted by Arden and Constable on electroretinography, which demonstrated that light stimulation generates a potential of approximately 6–10 mV [12]. This electrical activity may underlie the electromagnetic wave emissions hypothesized in our study. Moreover, the electromagnetic waves generated and propagated by the electrical activity of retinal cells exhibit functional similarities to piezoelectric crystals in radar or ultrasound devices, as described by Bachofer et al. [47]. Bachofer’s seminal study demonstrated that electromagnetic waves are produced in the retina when stimulated by X-rays, suggesting that the retina may respond to various forms of electromagnetic radiation, including, but not limited to, visible light, by generating electromagnetic waves. These findings provide further support for the hypothesis that the retina functions as a bidirectional electromagnetic transducer, contributing to a deeper understanding of its potential roles beyond light perception [47,48,49].

Building on these studies, our hypothesis can be summarized as follows: When light from an object stimulates the retina and the occipital cortex, the object is initially perceived. We propose that electromagnetic waves, generated by electrical activity in the retina stimulated by light, are emitted from the retina, which acts similarly to a dish antenna. These waves pass through the pupil, interact with objects in the external environment, and reflect back. The retina evaluates the phase difference and energy loss of these emitted and reflected waves, analogous to a radar mechanism, enabling the perception of the distance, shape, and depth of objects. The retina, in conjunction with the occipital cortex, functions similarly to the cache system in computers, facilitating the initial recognition of objects (Figure 2). This relationship suggests that the eyes are not merely passive receptors but may also actively transmit electromagnetic waves. Such a mechanism parallels the operation of an antenna in radar systems, enabling the eyes to discern the position and distance of surrounding objects. These findings lend further support to our hypothesis that the eyes might employ a radar- or ultrasound-like mechanism for depth and distance perception. Nevertheless, additional molecular and electrophysiological studies are required to elucidate the precise mechanisms underlying this process and its contribution to visual perception.

The findings of our study may complement existing theories on the functioning of the visual system. While the role of binocular vision in depth and distance perception is well established [50], the ability to perceive depth with a single eye highlights the presence of alternative mechanisms [51,52,53]. The radar- or ultrasound-like mechanism we propose may provide insights into how monocular depth cues are processed. Furthermore, our hypothesis raises intriguing questions about the role of the visual cortex. The suggestion that the occipital cortex might function as a radar or ultrasound detector necessitates a re-evaluation of how visual information is processed. In this context, Livingstone and Hubel’s seminal work on parallel processing pathways in the visual cortex offers a framework that aligns with our hypothesis [54]. Additionally, our hypothesis aligns with the framework proposed by Marr [55], who suggested that visual perception can be analyzed at three levels: (1) the computational level, (2) the algorithmic level, and (3) the implementation level.

Our work presents several potential real-world applications. For instance, it may inspire novel strategies for the design of retinal prostheses. Current research on retinal implants seeks to stimulate retinal nerves through electrical impulses to evoke the perception of a point in space [56]. The radar- or ultrasound-like mechanism we propose could inform the development of new approaches and designs for such devices. Additionally, our findings may enhance the understanding of the pathophysiology of certain visual disorders. For example, the loss of retinal ganglion cells, as seen in glaucoma patients [57], can be understood not only in the context of visual field defects but also as a disruption of the proposed radar- or ultrasound-like mechanism. This loss not only impairs the transmission of visual signals but also affects the generation and modulation of electromagnetic signals, which may explain the observed deficits in depth perception. Similarly, retinal vascular diseases such as diabetic retinopathy offer new insights into how vascular damage impacts depth perception. The altered metabolism of the RPE due to vascular damage may disrupt biophoton emission and electromagnetic signal modulation, further impairing visual function.

Future research could include in vitro studies aimed at measuring electromagnetic wave emissions from retinal cells using more sensitive techniques. In this context, researchers employed advanced electrophysiological methods that could be adapted for this purpose [58,59]. Additionally, brain imaging techniques such as functional magnetic resonance imaging and magnetoencephalography could be utilized to investigate the cortical consequences of the proposed mechanism [60]. Psychophysical experiments could also be designed to examine the behavioral implications of the radar- or ultrasound-like mechanism. This approach could extend and refine the findings of Westheimer on depth perception [61,62]. Our hypothesis has the potential to inspire biomimetic applications [63], as studying the radar- or ultrasound-like functioning of the eye may lead to the development of advanced imaging systems and sensors.

**Figure 3:**
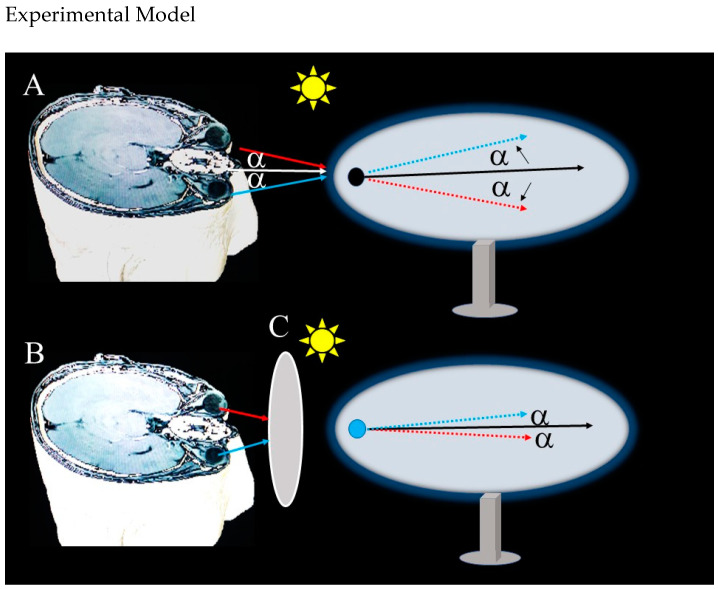
This model will investigate the propagation of electromagnetic waves (6–10 mV) from the human retina in a shielded, electronics-free room, using a magnet–vacuum setup with an evacuated ellipsoid tube (1 cm diameter, 4 cm long) and a magnetic needle (0.3 mm thick, 4 cm long) to detect directional changes (alpha angle) in these waves during eye movements by volunteers with normal vision. The eyes will be closed for 3 min, then opened in an illuminated environment, and horizontal/vertical eye movements will be recorded, showing that the magnetic needle will deflect with a varying alpha angle, suggesting wave propagation from the retina (**A**). A control condition, placing a wooden board between the eyes and the tube (**B**), will result in reduced or absent deflection, confirming the retinal origin of the waves (**C**). These preliminary findings, presented as a framework for future studies pending ethical approval, will offer initial evidence that electromagnetic waves emitted and absorbed by the retina could serve as a radar-like signal channel for spatial perception.

To further validate our hypothesis and address the limitations identified, we propose a preliminary experimental model (Figure 3), detailed in the Section 4, which will investigate the propagation of electromagnetic waves (6–10 mV) from the human retina using a magnet–vacuum setup in a shielded environment. This model, involving an evacuated ellipsoid tube and a magnetic needle to detect directional changes (alpha angle) during eye movements by volunteers, will offer initial evidence for the radar-like function of the eye in depth and distance perception. Although this model is theoretical and will await ethical approval for human application, it will provide a robust framework for future hypothesis-driven research, enhancing our understanding of the electromagnetic properties of the visual system and guiding investigations into its role in spatial awareness.

However, our study has several limitations. The data are primarily derived from animal models, and direct applicability to the human eye requires further investigation. While our findings are promising, larger, controlled studies in humans are essential for validation. Furthermore, more detailed investigations into the neural mechanisms underlying the proposed radar- or ultrasound-like system are needed. Advanced techniques, such as electrophysiological and neuroanatomical studies, combined with computational models simulating eye function, could provide deeper insights into the nature and operation of this mechanism.

## 5. Conclusions

In conclusion, this study proposes a novel hypothesis for the functioning of the eyes, suggesting that the retina’s complex, layered structure, with each layer potentially exhibiting distinct electromagnetic properties, may enable the eye to function as a radar- or ultrasound-like system. Combined with the observed electro-oculographic correlations, our findings indicate that the mechanisms underlying depth and distance perception may be more intricate than previously assumed. This perspective opens new avenues for research in visual physiology and neuroscience, offering deeper insights into the processes governing visual perception. If validated, this hypothesis could revolutionize vision science and neuro-ophthalmology, providing a foundation for new diagnostic and therapeutic advancements. Future studies should prioritize the validation of this hypothesis and undertake detailed investigations into the electromagnetic properties of the visual system.

## Data Availability

The original contributions presented in this study are included in the article; further inquiries can be directed to the corresponding author.
